# Association of a Dietary Index for Gut Microbiota With Epilepsy Among US Adults Aged 40 and Over: A Cross‐Sectional Study

**DOI:** 10.1155/bn/5311940

**Published:** 2026-09-15

**Authors:** Jialin Yu, Hailong Li, Hongmei Zeng

**Affiliations:** ^1^ Department of Neurology, The Second Affiliated Hospital, Jiangxi Medical College, Nanchang University, Nanchang, Jiangxi, China, ncu.edu.cn; ^2^ Department of Cardiovascular Medicine, The Second Affiliated Hospital, Jiangxi Medical College, Nanchang University, Nanchang, Jiangxi, China, ncu.edu.cn

**Keywords:** dietary index for gut microbiota, dietary patterns, epilepsy, gut microbiota, NHANES

## Abstract

**Background:**

Epilepsy remains difficult to control in some patients, motivating interest in modifiable factors linked to seizure biology. Because diet can shape gut microbial ecology and gut–brain signaling, we examined whether the dietary index for gut microbiota (DI‐GM) was associated with prevalent epilepsy in US adults aged ≥ 40 years.

**Methods:**

This cross‐sectional study included 5025 participants from NHANES 2013–2016. DI‐GM was constructed from 14 predefined dietary components reflecting microbiota‐supportive or microbiota‐adverse intake. NHANES sampling weights were applied. Multivariable logistic regression evaluated associations between DI‐GM and prevalent epilepsy. Restricted cubic splines, subgroup analyses, and weighted quantile sum (WQS) regression were used to examine dose–response shape, effect heterogeneity, and component contributions.

**Results:**

In the fully adjusted model, DI‐GM showed an inverse association with prevalent epilepsy (OR per 1‐point increase, 0.80; 95% CI, 0.69–0.93; *p* = 0.008). Compared with Q1, Q4 had lower epilepsy odds (OR, 0.29; 95% CI, 0.10–0.87; *p* = 0.033). The spline analysis did not support nonlinearity (*p* = 0.663). The inverse association was mainly seen among adults without hypertension or diabetes, with significant interaction by hypertension (*p* < 0.001) and diabetes status (*p* = 0.003). WQS results highlighted cranberries, chickpeas, avocados, soybeans, and lower processed meat intake as major contributors.

**Conclusions:**

Higher DI‐GM adherence was associated with lower odds of prevalent epilepsy, particularly in metabolically healthier subgroups. Prospective and intervention studies are needed to test temporality and clinical relevance.

## 1. Introduction

Epilepsy represents a major neurological condition in which individuals have an enduring susceptibility to unprovoked seizures, leading to substantial health‐related and socioeconomic consequences [1]. Despite the widespread use of antiseizure medications (ASMs), pharmacological therapy does not provide adequate seizure control for all individuals with epilepsy [2–4]. In addition, adverse cognitive, behavioral, and mood‐related effects of ASMs can limit long‐term treatment tolerance [5]. These challenges have increased interest in modifiable lifestyle exposures, including diet, that may influence seizure susceptibility or the broader neurological milieu.

The gut microbiota is increasingly regarded as a biological interface linking intestinal homeostasis with systemic physiology and brain function, especially through neuroimmune and metabolic signaling pathways [6, 7]. Communication between the intestine and the brain is not mediated by a single pathway but instead involves immune responses, endocrine factors, microbial metabolites, and neural circuits, which together constitute the gut–brain axis. In the context of epilepsy, disturbances in gut microbial ecology have been associated with systemic inflammatory activation, altered GABA‐ and glutamate‐related metabolism, and impaired barrier integrity, mechanisms that may plausibly influence seizure vulnerability [8–10]. Evidence from experimental models and clinical studies further suggests that ketogenic diet therapy may modulate seizure activity partly through pathways involving gut microbial remodeling [11–14]. However, most available evidence has focused on ketogenic interventions or pediatric populations, whereas the relevance of broader habitual dietary patterns in adults remains insufficiently characterized.

Diet is one of the strongest environmental influences on gut microbial composition and metabolic activity [15]. Microbiota‐supportive diets, characterized by higher intake of fiber, polyphenols, legumes, fermented foods, and plant‐derived substrates, may favor short‐chain fatty acid (SCFA)–producing microbial activity and influence inflammatory and neuroprotective mechanisms associated with epilepsy [16–18]. By comparison, intake patterns rich in refined grains, red and processed meats, and fat‐dense foods may be associated with gut dysbiosis and systemic low‐grade inflammation. The dietary index for gut microbiota (DI‐GM), proposed by Kase et al. [19], summarizes intake of microbiota‐relevant dietary components and provides a practical exposure metric for population‐based studies. Assessing the DI‐GM in relation to epilepsy is therefore biologically plausible, particularly among adults aged 40 years and older, in whom acquired epilepsy and cardiometabolic comorbidities become more common.

Population‐based evidence on microbiota‐oriented dietary indices and epilepsy in middle‐aged and older adults is limited. Using data from US adults aged 40 years and older, we aimed to clarify the relationship between DI‐GM and prevalent epilepsy.

## 2. Methods

### 2.1. Study Population

This study was based on the 2013–2016 National Health and Nutrition Examination Survey (NHANES), a publicly available survey of the US population. The original NHANES protocol was approved by the National Center for Health Statistics Research Ethics Review Board, and written informed consent was collected from all participants before survey participation.

**Figure 1:**
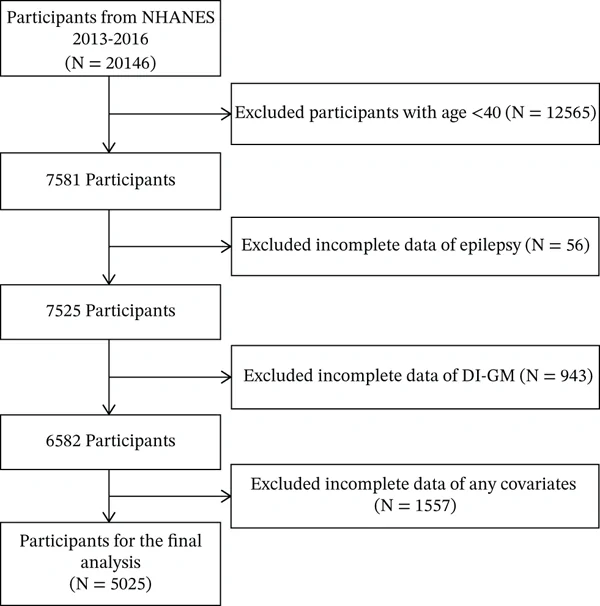
Flow diagram of study cohort selection.

The source population included 20,146 interviewed respondents. For the present analysis, eligibility was restricted to adults aged 40 years or older with available information on epilepsy status, DI‐GM assessment, and covariates required for the fully adjusted model. We excluded 12,565 participants younger than 40 years, 56 participants without epilepsy status information, 943 participants with incomplete DI‐GM data, and 1557 participants with missing covariate values. After these exclusions, 5025 participants were included in the final analytic sample (Figure 1).

### 2.2. Assessment of DI‐GM

The DI‐GM was derived using the scoring method proposed by Kase et al. [19]. The DI‐GM consists of 14 dietary components, including components considered supportive of gut microbial health and components considered unfavorable to gut microbial health. Component definitions, sex‐specific median cutoffs, and scoring rules are provided in Table S1.

Dietary intake was assessed from 24‐h dietary recall data collected during the 2013–2016 NHANES cycles. Each DI‐GM component contributed either 0 or 1 point. For microbiota‐supportive components, intake at or above the sex‐specific median was assigned 1 point. For microbiota‐unfavorable components, intake below the sex‐specific median was assigned 1 point, except for the high‐fat diet component. For this component, 1 point was assigned when fat contributed less than 40% of total energy intake. The 14 component scores were added to generate the total DI‐GM score, ranging from 0 to 14, with higher values indicating a more microbiota‐favorable dietary pattern. Based on survey‐weighted quartile cutoffs, DI‐GM was categorized as Q1, 0–3 points; Q2, 4 points; Q3, 5 points; and Q4, 6–14 points. The distribution of DI‐GM scores according to sex is presented in Table S3.

### 2.3. Diagnosis of Epilepsy

Epilepsy case status was defined using information from the NHANES Prescription Medications section, in which participants reported prescription medications taken during the 30 days before the interview. Participants were considered to have epilepsy when manual review confirmed prescription medication use associated with ICD‐10 Code G40. The frequency of ASMs contributing to the epilepsy case definition is presented in Table S2. This approach to epilepsy ascertainment was adapted from previous studies using NHANES medication data [20].

### 2.4. Covariate Assessment

To account for factors that may be related to both dietary pattern and epilepsy status, we included a prespecified set of covariates from NHANES questionnaire and examination data. These variables covered sociodemographic characteristics, health behaviors, economic status, cardiometabolic comorbidities, overall adiposity, energy intake, and depression. The final multivariable models included age (< 70 or ≥ 70 years), sex, race/ethnicity, education, marital status, smoking, alcohol consumption, poverty‐to‐income ratio (PIR), hypertension, diabetes mellitus (DM), cardiovascular disease (CVD), body mass index (BMI), total energy intake, and depression status.

### 2.5. Statistical Analysis

All analyses accounted for the NHANES complex survey design by incorporating sampling weights, strata, and primary sampling units. Continuous variables were summarized as weighted means with standard errors, and categorical variables as counts with weighted percentages. Baseline characteristics were compared by epilepsy status using survey‐appropriate tests. The association between DI‐GM and prevalent epilepsy was evaluated using survey‐weighted multivariable logistic regression. DI‐GM was examined as both a continuous score and quartile‐based categories, with the lowest quartile as the reference. Results were expressed as odds ratios (ORs) and 95% confidence intervals (CIs). Restricted cubic spline models were fitted within the fully adjusted model to evaluate whether the DI‐GM–epilepsy association departed from linearity, with knots placed at the 25th, 50th, and 75th percentiles of DI‐GM. Effect modification was explored in subgroup analyses stratified by age, sex, educational attainment, marital status, PIR, smoking status, depression, hypertension, diabetes mellitus, and cardiovascular disease; interaction *p* values were obtained using likelihood ratio tests. Weighted quantile sum (WQS) regression was used to assess the combined contribution of the 14 DI‐GM components and their relative weights. Statistical significance was defined as a two‐sided *p* value < 0.05. Analyses were performed using R Version 4.4.2.

## 3. Results

### 3.1. Baseline Characteristics

The final study sample consisted of 5025 NHANES participants enrolled between 2013 and 2016. After survey weighting, the mean age was 58.26 years, and 52.22% of participants were female. Table 1 provides the weighted distribution of baseline variables.

**Table 1:** Weighted characteristics of the study population.

Characteristics	Total	Nonepilepsy	Epilepsy	*p* value
Number	5025	4974	51	
Age (years)	58.26 (0.34)	58.25 (0.34)	59.96 (2.01)	0.391
Sex (%)				0.944
Male	2386 (47.78)	2361 (47.79)	25 (47.01)	
Female	2639 (52.22)	2613 (52.21)	26 (52.99)	
Race (%)				0.759
Non‐Hispanic White	2249 (72.53)	2223 (72.54)	26 (70.87)	
Non‐Hispanic Black	1027 (9.47)	1018 (9.45)	9 (11.66)	
Mexican American	683 (6.38)	678 (6.36)	5 (8.66)	
Other race	1066 (11.62)	1055 (11.65)	11 (8.81)	
Education level (%)				0.034
High school or less	2179 (33.79)	2156 (33.64)	23 (51.48)	
More than high school	2846 (66.21)	2818 (66.36)	28 (48.52)	
Marital status (%)				0.010
Married or living with partner	3172 (68.46)	3148 (68.58)	24 (54.27)	
Living alone	1853 (31.54)	1826 (31.42)	27 (45.73)	
PIR (%)				0.016
Low	944 (11.25)	930 (11.17)	14 (20.49)	
Middle	2675 (46.80)	2642 (46.64)	33 (66.42)	
High	1406 (41.95)	1402 (42.19)	4 (13.09)	
Smoking status (%)				0.926
Never	2631 (52.73)	2607 (52.75)	24 (50.18)	
Now	871 (16.43)	858 (16.41)	13 (18.05)	
Former	1523 (30.85)	1509 (30.84)	14 (31.77)	
Drinking status (%)				0.116
Never	736 (11.13)	727 (11.10)	9 (14.77)	
Mild	1853 (40.56)	1843 (40.73)	10 (19.19)	
Moderate	707 (16.91)	701 (16.94)	6 (13.70)	
Heavy	655 (13.56)	648 (13.54)	7 (15.03)	
Former	1074 (17.85)	1055 (17.69)	19 (37.31)	
Depression (%)				< 0.001
No	4550 (91.33)	4512 (91.49)	38 (71.77)	
Yes	475 (8.67)	462 (8.51)	13 (28.23)	
Hypertension (%)				0.840
No	2197 (48.63)	2176 (48.62)	21 (50.08)	
Yes	2828 (51.37)	2798 (51.38)	30 (49.92)	
DM (%)				0.768
No	3708 (79.46)	3671 (79.45)	37 (80.79)	
Yes	1317 (20.54)	1303 (20.55)	14 (19.21)	
CVD (%)				< 0.001
No	4258 (86.84)	4226 (87.00)	32 (66.72)	
Yes	767 (13.16)	748 (13.00)	19 (33.28)	
Energy intake (kcal)	2051.97 (15.64)	2052.10 (15.65)	2035.77 (168.04)	0.923
BMI (kg/m^2^)	29.72 (0.19)	29.71 (0.19)	30.68 (1.33)	0.483
DI‐GM	5.18 (0.05)	5.19 (0.05)	4.45 (0.18)	< 0.001

*Note:* Presentation of continuous variables was as mean (standardized errors). Presentation of categorical variables was as number (weighted percentage).

Abbreviations: BMI, body mass index; CVD, cardiovascular disease; DI‐GM, dietary index for gut microbiota; DM, diabetes mellitus; PIR, poverty‐to‐income ratio.

Relative to participants without epilepsy, those with epilepsy showed a less favorable socioeconomic and clinical profile. They were more likely to have high school education or lower (51.48% vs. 33.64%; *p* = 0.034), to live alone (45.73% vs. 31.42%; *p* = 0.010), to have a low PIR (20.49% vs. 11.17%; *p* = 0.016), to report depression (28.23% vs. 8.51%; *p* < 0.001), and to have CVD (33.28% vs. 13.00%; *p* < 0.001). DI‐GM also differed by epilepsy status, with lower weighted mean scores in the epilepsy group than in the nonepilepsy group (4.45 ± 0.18 vs. 5.19 ± 0.05; *p* < 0.001). Other baseline variables, including age, sex, race/ethnicity, smoking, alcohol use, hypertension, DM, energy intake, and BMI, were comparable between groups.

### 3.2. DI‐GM and Prevalent Epilepsy

The weighted regression results are summarized in Table 2. In the continuous score analysis, DI‐GM was negatively associated with prevalent epilepsy after full adjustment, with each 1‐point increase corresponding to a lower odds estimate (Model 3: OR, 0.80; 95% CI, 0.69–0.93; *p* = 0.008). Estimates from the unadjusted and partially adjusted models showed the same direction of association.

**Table 2:** Weighted logistic regression analysis of the association between DI‐GM and epilepsy.

Characteristic	Model 1	Model 2	Model 3
	OR (95% CI), *p* value	OR (95% CI), *p* value	OR (95% CI), *p* value
DI‐GM (continuous)	0.77 (0.67, 0.89), < 0.001	0.80 (0.70, 0.92), 0.003	0.80 (0.69, 0.93), 0.008
DI‐GM (categorical)			
Q1 (≤ 3)	Reference	Reference	Reference
Q2 (4)	1.28 (0.49, 3.34), 0.601	1.40 (0.51, 3.86), 0.496	1.33 (0.40, 4.43), 0.578
Q3 (5)	0.50 (0.18, 1.41), 0.183	0.56 (0.19, 1.64), 0.273	0.58 (0.15, 2.20), 0.353
Q4 (≥ 6)	0.25 (0.10, 0.67), 0.007	0.29 (0.11, 0.77), 0.015	0.29 (0.10, 0.87), 0.033
*p* for trend	0.002	0.009	0.026

*Note:* Model 1, unadjusted; Model 2, adjusted for age, sex, race, educational level, and marital status; Model 3, adjusted for age, sex, race, education level, marital status, BMI, PIR, smoking status, drinking status, energy intake, CVD, DM, depression, and hypertension.

Abbreviations: BMI, body mass index; CI, confidence interval; CVD, cardiovascular disease; DI‐GM, dietary index for gut microbiota; DM, diabetes mellitus; OR, odds ratio; PIR, poverty‐to‐income ratio; Q, quantile.

When DI‐GM was analyzed by quartiles, participants in the highest category had lower odds of epilepsy than those in Q1 in the fully adjusted model. The estimates for Q2 and Q3 did not reach statistical significance. A significant trend across increasing DI‐GM categories was observed in Model 3 (*p* for trend = 0.026).

### 3.3. Nonlinear Association Analysis

**Figure 2:**
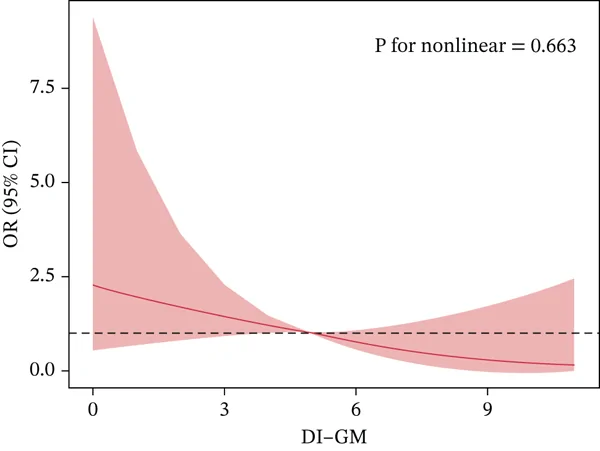
Association of DI‐GM with epilepsy by RCS analysis.

Using the fully adjusted covariate set, restricted cubic spline modeling did not identify a nonlinear DI‐GM–epilepsy relationship (*p* for nonlinearity = 0.663). The spline results therefore supported an approximately linear decline in epilepsy odds across the observed DI‐GM distribution (Figure 2).

### 3.4. Subgroup Analyses and Tests for Effect Modification

Using continuous DI‐GM in the fully adjusted model, we explored whether the DI‐GM–epilepsy association differed across participant subgroups (Table 3). Significant interaction tests were found for hypertension and DM, with corresponding *p* values of < 0.001 and 0.003. In the nonhypertensive subgroup, DI‐GM showed an inverse association with prevalent epilepsy (OR, 0.55; 95% CI, 0.39–0.77), whereas no clear association was seen among participants with hypertension (OR, 1.10; 95% CI, 0.91–1.32). For DM, the inverse association was limited to participants without DM (OR, 0.70; 95% CI, 0.58–0.84) and was not observed in those with DM (OR, 1.29; 95% CI, 0.89–1.87).

**Table 3:** Exploratory subgroup analyses of the association between DI‐GM and epilepsy.

	OR (95% CI)	*p* value	*p* for interaction
Age			0.392
< 70	0.74 (0.60, 0.92)	0.013	
≥ 70	0.91 (0.70, 1.20)	0.470	
Sex			0.926
Male	0.77 (0.56, 1.05)	0.092	
Female	0.78 (0.56, 1.10)	0.136	
Educational attainment			0.262
High school or less	0.85 (0.69, 1.04)	0.107	
More than high school	0.73 (0.54, 1.00)	0.051	
Marital status			0.201
Married or living with partner	0.86 (0.66, 1.11)	0.203	
Living alone	0.71 (0.52, 0.98)	0.038	
PIR			0.674
Low	0.74 (0.40, 1.37)	0.303	
Middle	0.76 (0.59, 0.98)	0.038	
High	0.81 (0.46, 1.42)	0.416	
Smoking status			0.343
Never	0.67 (0.50, 0.91)	0.014	
Former	1.02 (0.57, 1.83)	0.937	
Now	0.83 (0.64, 1.07)	0.128	
Depression			0.341
No	0.86 (0.71, 1.04)	0.109	
Yes	0.52 (0.24, 1.14)	0.090	
Hypertension			< 0.001
No	0.55 (0.39, 0.77)	0.003	
Yes	1.10 (0.91, 1.32)	0.292	
DM			0.003
No	0.70 (0.58, 0.84)	0.002	
Yes	1.29 (0.89, 1.87)	0.158	
CVD			0.463
No	0.76 (0.61, 0.94)	0.018	
Yes	0.86 (0.68, 1.08)	0.171	

*Note:* Adjusted for age, sex, race, education level, marital status, BMI, PIR, smoking status, drinking status, energy intake, CVD, DM, depression, and hypertension.

Abbreviations: BMI, body mass index; CI, confidence interval; CVD, cardiovascular disease; DI‐GM, dietary index for gut microbiota; DM, diabetes mellitus; OR, odds ratio; PIR, poverty‐to‐income ratio.

### 3.5. WQS Regression

**Figure 3:**
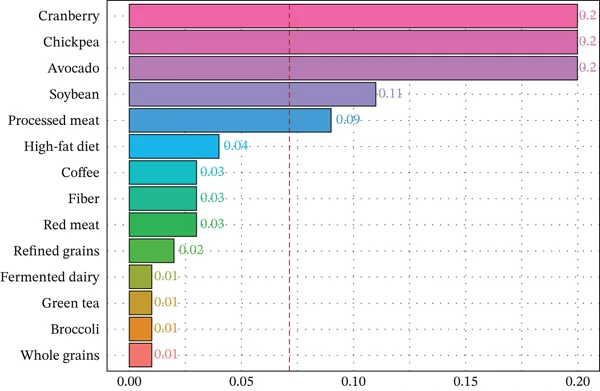
Weighted quantile sum (WQS) regression weights for DI‐GM components in relation to epilepsy, adjusted for age, sex, race, education, marital status, BMI, PIR, smoking status, drinking status, energy intake, CVD, DM, depression, and hypertension.

After adjustment for Model 3 covariates, WQS regression was applied to assess the joint contribution of DI‐GM components to epilepsy odds (Figure 3). The negative direction WQS analysis identified cranberry, chickpea, avocado, and soybean as the main contributors to the index, accounting for weights of 0.20, 0.20, 0.20, and 0.11, respectively. Processed meat also contributed meaningfully to the inverse mixture index (weight = 0.09), reflecting the scoring of lower processed meat intake as favorable. Whole grains, broccoli, fermented dairy products, and green tea had the smallest weights (0.01 each).

## 4. Discussion

This study provides population‐based evidence that microbiota‐oriented diet quality may be related to epilepsy status in adults. This pattern was observed when DI‐GM was evaluated either continuously or by category, and it was not fully explained by the demographic, socioeconomic, lifestyle, cardiometabolic, or mental health factors included in the models. These findings place microbiota‐oriented diet quality as a potential epidemiological correlate of epilepsy in adults. Because DI‐GM and epilepsy status were assessed at the same survey period, the results should be interpreted as cross‐sectional associations rather than evidence that diet alters epilepsy risk.

Several biological pathways may underlie this association. The gut–brain axis provides a plausible framework linking diet, microbial metabolism, immune signaling, and neuronal excitability [9, 21]. Changes in food consumption are capable of inducing marked shifts in gut microbial profiles and metabolic capacity over short time frames [15, 22]. DI‐GM components such as legumes, fiber‐rich foods, cranberries, avocados, broccoli, coffee, and tea provide fermentable substrates or phytochemicals that may support beneficial microbial taxa and SCFA production [23–25]. SCFAs, particularly butyrate, can strengthen gut barrier integrity, regulate systemic and neuroinflammatory responses, and influence neurotransmitter‐related pathways, all of which are relevant to epileptogenesis and seizure susceptibility [16–18]. By contrast, diets dominated by processed products, red meat, refined carbohydrates, and excessive fat may contribute to microbial imbalance, increased gut permeability, and activation of inflammatory pathways [26]. Therefore, the observed inverse association is consistent with, but does not prove, a microbiota‐mediated diet–epilepsy pathway.

Findings from the WQS model suggested that several dietary components contributed jointly to the DI‐GM–epilepsy association, with no single component fully accounting for the pattern. Cranberries, chickpeas, avocados, and soybeans carried the largest weights in the favorable mixture index. These foods contain polyphenols, fiber, unsaturated fats, and other bioactive compounds that may influence microbial metabolism [27–29]. The contribution of processed meat is also informative, because lower processed meat intake was scored as favorable and contributed to the overall negative mixture association. Components with small weights, such as whole grains, broccoli, fermented dairy products, coffee, and green tea, should not be interpreted as biologically unimportant; their lower weights may reflect limited intake variability, correlations among components, measurement error, or the constraints of mixture modeling. Overall, the results favor interpretation of DI‐GM as an integrated dietary pattern rather than as a proxy for a single food item.

A notable finding was the apparent modification of the DI‐GM–epilepsy association by hypertension and DM. The inverse association was present among participants without these conditions, whereas it was not evident among those with hypertension or DM. This pattern should be interpreted cautiously because subgroup analyses are exploratory and the number of epilepsy cases was limited. These cardiometabolic disorders can reshape the host biological environment through sustained inflammation, endothelial injury, disturbed metabolic homeostasis, and alterations in intestinal microbial ecology [30–33]. They are also linked to vascular brain injury and late‐onset epilepsy risk [34, 35]. In individuals with these comorbidities, vascular and metabolic mechanisms, medication effects, or differences in epilepsy etiology may attenuate or mask the association between general diet quality and epilepsy [36]. Therefore, assessment of diet–neurology associations should incorporate cardiometabolic comorbidity profiles as a potentially important modifying factor.

The clinical implications of these findings should remain conservative. Our results add to evidence that dietary modulation may be relevant to neurological health beyond specialized ketogenic diet protocols, which are often used in selected epilepsy populations. A diet pattern emphasizing diverse plant‐based foods while limiting processed and high‐fat items may be easier to implement at the population level. However, the present study cannot establish whether improving DI‐GM would prevent epilepsy or reduce seizure burden. Reverse causation is possible, because epilepsy, ASMs, or related comorbidities may influence appetite, food choices, or dietary restrictions. Prospective and interventional studies are therefore required before DI‐GM‐based dietary recommendations can be incorporated into epilepsy prevention or management strategies.

A major strength of this study is the use of NHANES, which includes a large sample and standardized assessment procedures, thereby improving the relevance of our findings to US adults aged at least 40 years. The DI‐GM offers a biologically informed index focused on diet–microbiota interactions. We also applied multiple complementary analyses, including survey‐weighted regression, RCS modeling, subgroup interaction testing, and WQS regression to evaluate component‐level contributions.

The findings should be interpreted in light of several limitations. First, the cross‐sectional nature of the study limits our ability to establish whether DI‐GM preceded epilepsy status and precludes causal inference. Second, epilepsy was identified through recent prescription medication use for ICD‐10 Code G40; this approach may misclassify some participants and does not capture epilepsy type, seizure frequency, duration, or severity. Third, only 51 participants met the epilepsy definition, which limits statistical power, widens CIs, and may reduce model stability, especially in subgroup analyses. Fourth, the use of 24‐h dietary recalls may not adequately reflect habitual dietary intake and could introduce errors related to memory, reporting, and dietary measurement. Fifth, residual confounding remains possible despite multivariable adjustment, including confounding by genetics, medication classes, neurological history, and unmeasured lifestyle factors. Finally, because DI‐GM is based on dietary intake data, it should be regarded as a surrogate exposure indicator and not as a direct assessment of gut microbiome structure or function.

Given these limitations, the present work should be viewed as an exploratory study generating hypotheses for future research. Prospective cohorts with repeated dietary assessment, detailed epilepsy phenotyping, medication information, and microbiome profiling are needed to test temporality and clarify mechanisms. Metagenomic and metabolomic analyses could help identify microbial taxa, functional pathways, and metabolites linking dietary patterns with epilepsy risk. Ultimately, randomized dietary intervention studies will be necessary to determine whether modifying microbiota‐oriented dietary patterns can influence epilepsy incidence or clinical outcomes.

## 5. Conclusions

In this NHANES‐based analysis, higher DI‐GM scores identified a dietary pattern that was less frequently observed among adults with prevalent epilepsy. The inverse pattern was approximately linear and appeared stronger in participants free of hypertension or DM, pointing to possible modification by cardiometabolic health. These results add epidemiological support to a potential diet–microbiota–epilepsy link, but the cross‐sectional design does not allow temporal or causal conclusions. Prospective cohorts and randomized dietary trials are required before microbiota‐oriented dietary strategies can be considered relevant to epilepsy prevention or clinical management.

## Author Contributions

Jialin Yu contributed to conceptualization, formal analysis, methodology, software, and writing of the original draft. Hailong Li contributed to conceptualization, formal analysis, data curation, methodology, and writing of the original draft. Hongmei Zeng contributed to conceptualization, supervision, validation, writing—review and editing, and project administration. Jialin Yu and Hailong Li made equal contributions to this work and share the first authorship.

## Funding

No funding was received for this manuscript.

## Conflicts of Interest

The authors declare no conflicts of interest.

## Supporting information


**Supporting Information** Additional supporting information can be found online in the Supporting Information section. The supporting information associated with this article includes the following tables. Table S1: The scoring details for each component in DI‐GM. Table S2: Frequency of each ASM with indication for treating seizures, counting toward our case definition of epilepsy. Table S3: Distribution of DI‐GM scores among female and male participants included in the study.

## Data Availability

The datasets analyzed in this study were obtained from NHANES and are publicly available from the NCHS website: https://wwwn.cdc.gov/nchs/nhanes/Default.aspx.
