# Supplementary material for: Association of a Dietary Index for Gut Microbiota With Epilepsy Among US Adults Aged 40 and Over: A Cross‐Sectional Study
Source: Behav Neurol. 2026 Sep 15;2026:5311940. doi: 10.1155/bn/5311940 (PMC13575649; doi:10.1155/bn/5311940)
Supplement: Supplementary file 1 [file BN-2026-5311940-s001.docx]

**Table S1. The scoring details for each component in DI-GM**

| Component | Female median | Male median |
| --- | --- | --- |
| Avocado | 0 | 0 |
| Broccoli | 0 | 0 |
| Chickpea | 0.0000 | 0.0000 |
| Coffee | 255 | 300 |
| Cranberry | 0 | 0 |
| Fermented dairy | 241 | 258 |
| Fiber | 14 | 17 |
| Green tea | 0 | 0 |
| Soybean | 0 | 0 |
| Whole grains | 0.27 | 0.28 |
| Fat | 63 | 82 |
| Refined grains | 4.1 | 5.2 |
| Processed meat | 0.00 | 0.00 |
| Red meat | 1.02 | 1.94 |

**Table S2. Frequency of each ASM with indication for treating seizures, counting towards our case definition of epilepsy.**

| Antiseizure medication | Frequency |
| --- | --- |
| Phenytoin | 14 |
| Levetiracetam | 11 |
| Gabapentin | 9 |
| Lamotrigine | 8 |
| Divalproex | 6 |
| Carbamazepine | 5 |
| Oxcarbazepine | 3 |
| Topiramate | 3 |
| Phenobarbital | 3 |
| Clonazepam | 2 |
| Primidone | 2 |
| Diazepam | 2 |
| Alprazolam | 1 |
| Pregabalin | 1 |
| Clobazam | 1 |

**Table S3 Distribution of DI-GM scores according to sex**

| DI_GM score | Female participants | Female proportions | Male participants | Male proportions |
| --- | --- | --- | --- | --- |
| 0 | 3 | 0.11 | 2 | 0.08 |
| 1 | 31 | 1.17 | 37 | 1.55 |
| 2 | 110 | 4.17 | 139 | 5.83 |
| 3 | 300 | 11.37 | 292 | 12.24 |
| 4 | 539 | 20.42 | 506 | 21.21 |
| 5 | 620 | 23.49 | 554 | 23.22 |
| 6 | 516 | 19.55 | 425 | 17.81 |
| 7 | 305 | 11.56 | 268 | 11.23 |
| 8 | 142 | 5.38 | 119 | 4.99 |
| 9 | 52 | 1.97 | 34 | 1.42 |
| 10 | 17 | 0.64 | 10 | 0.42 |
| 11 | 4 | 0.15 |  |  |
